# Study on bending behaviour of nickel–titanium rotary endodontic instruments by analytical and numerical analyses

**DOI:** 10.1111/iej.12025

**Published:** 2012-11-22

**Authors:** C C Tsao, J U Liou, P H Wen, C C Peng, T S Liu

**Affiliations:** 1 Department of Mechanical Engineering, National Chiao Tung UniversityHsinchu, Taiwan; 2 Department of Dentistry, Division of Endodontics, Mackay Memorial HospitalTaipei, Taiwan

**Keywords:** bending, endodontic instruments, Euler–Bernoulli beam, finite element method, large deformation

## Abstract

**Aim:**

To develop analytical models and analyse the stress distribution and flexibility of nickel–titanium (NiTi) instruments subject to bending forces.

**Methodology:**

The analytical method was used to analyse the behaviours of NiTi instruments under bending forces. Two NiTi instruments (RaCe and Mani NRT) with different cross-sections and geometries were considered. Analytical results were derived using Euler–Bernoulli nonlinear differential equations that took into account the screw pitch variation of these NiTi instruments. In addition, the nonlinear deformation analysis based on the analytical model and the finite element nonlinear analysis was carried out. Numerical results are obtained by carrying out a finite element method.

**Results:**

According to analytical results, the maximum curvature of the instrument occurs near the instrument tip. Results of the finite element analysis revealed that the position of maximum von Mises stress was near the instrument tip. Therefore, the proposed analytical model can be used to predict the position of maximum curvature in the instrument where fracture may occur. Finally, results of analytical and numerical models were compatible.

**Conclusion:**

The proposed analytical model was validated by numerical results in analysing bending deformation of NiTi instruments. The analytical model is useful in the design and analysis of instruments. The proposed theoretical model is effective in studying the flexibility of NiTi instruments. Compared with the finite element method, the analytical model can deal conveniently and effectively with the subject of bending behaviour of rotary NiTi endodontic instruments.

## Introduction

During root canal shaping, rotary nickel–titanium (NiTi) endodontic instrument are subjected to various forces. Fracture of such instruments may result from torsional overload or flexural fatigue or a combination of both (Camps & Pertot 1994). There have been reports of various factors that can affect the fracture of NiTi rotary files such as their geometric configuration (Yao *et al*. 2006), rotation rate and angles of root canal curvature (Martin *et al*. 2003). Fracture due to flexural fatigue (bending stress) occurs when a rotary endodontic instrument that has already been weakened by metal fatigue is placed under stress (Van der Vyver 2011). The fracture modes of rotary NiTi endodontic instruments especially fatigue have been investigated (Wei *et al*. 2007), and a wide range of fatigue life tests have been assessed (Kuhn & Jordan 2002, Di Fiore *et al*. 2006, Pirani *et al*. 2011).

To help analyse fracture mechanics, a numerical study using finite element methods on rotary NiTi endodontic instruments has reported that NiTi instruments perform better than stainless steel instruments (Lorenza *et al*. 2009). A model of three-dimensional rotary endodontic instruments has also been proposed (Chevalier *et al*. 2010). Finite element analysis was used to evaluate the bending fatigue lifetime of endodontic files by Cheung *et al*. (2011), who reported that NiTi and triangular geometry profiles have better fatigue resistance than stainless steel and square cross-sections. Mathematical and numerical models were applied to rotary endodontic instrument design during root canal preparation (Zhang *et al*. 2011), but the models were not compared. They reported that maximum stresses always appeared at the periphery of the instrument cross-section.

Higher bending and torsional stiffness characteristics of rotary endodontic instruments will enhance their cutting efficiency and reduce undesirable deformation (Kim *et al*. 2009). The bending and torsional stiffness characteristics of different cross-sections of rotary endodontic instruments were studied using the finite element method (Xu *et al*. 2006). According to the study, the instrument was more torque resistant when the area of the cross-section increased.

In addition to finite element analysis, analytical methods can deal conveniently and effectively with analysis of rotary endodontic instrument performance. Hence, this study investigates bending deformation and stress distribution of rotary endodontic instruments using finite element nonlinear analysis, and the nonlinear Euler–Bernoulli differential equation as an analytical alternative to numerical methods. Both results are compared. Accordingly, the flexibility and flexural failure of rotary NiTi endodontic instruments can be predicted.

## Material and methods

### Bending analysis for rotary NiTi endodontic instrument

A nonlinear model for analysing the bending behaviour of rotary NiTi endodontic instruments was derived using Euler–Bernoulli differential equations. When a moment *M* deforms a beam element, the strain at a position *y* from the neutral axis can be expressed by (Boresi & Schmidt 2003)


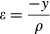
(1)

where *ɛ* is the strain. *ρ* is the radius of curvature. This equation shows that the strain is inversely proportional to the radius of curvature when a beam is subjected to bending. Hooke's law is written as (Boresi & Schmidt 2003)


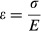
(2)

where *σ* is the stress, and *E* is the young's modulus. This equation shows that the strain is proportional to the stress. Fractures of instruments always occur at the same place with the maximum strain and stress. The Euler–Bernoulli law for solving large deformation problem of flexible beams that produced by bending can be written mathematically as (Fertis 2006)


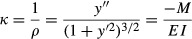
(3)

where *κ* is the curvature that causes by the bending moment, and it is the reciprocal of the radius of curvature. Moreover, the curvature is also associated with the deformed configuration of the instruments. *I* is the beam's moment of inertia computed about the neutral axis. *M* is the bending moment. The product *EI* in this equation is referred to as the flexural rigidity. Greater flexibility makes the instrument easier to pass along the curve of root canals. The Euler–Bernoulli law describes that the bending moment is proportional to the instrument curvature. When the loading and the cross-section moment of inertia vary with the *x*-axis, *I* and *M* are written as


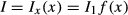
(4)


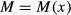
(5)

**Figure 1:**
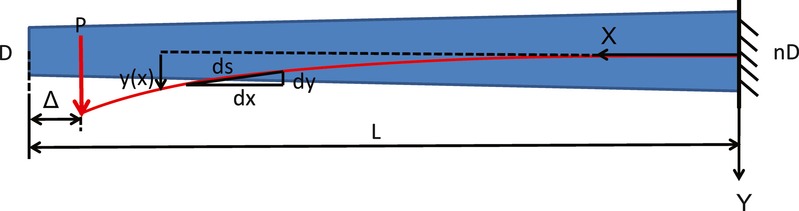
Cone-shape cantilever beam subjected to a concentrated load *P*.

*I*_1_ is the moment of inertia at the fixed end, and *f*(*x*) represents the variation with respect to *I*_1_. Substituting Eqns 4 and 5 into Eqn 3 yields a nonlinear differential equation that varies with the *x*-axis


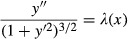
(6)

where 
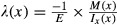
. For solving the nonlinear differential equation, the procedure may be complex, and the long series of calculation is described in Appendices S1 and S2. Figure 1 shows a cantilever beam of cone shape with a load *P* applied at the beam's free end and the bending moment can be expressed as


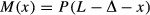
(7)

*L* denotes the beam length and it will not change after deformation, and Δ is the horizontal displacement of the tip. The bending moment is proportional to the load *P* and varies with the *x*-axis. For tapered beams with concentrated load only, the cross-section diameter varies along with the *x*-axis and the variation can be approximated by


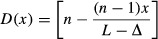
(8)

where *n* represents the ratio of maximum diameter versus minimum diameter. It represents the taper. The area moment of inertia with a circular cross-section is written as (Boresi & Schmidt 2003)


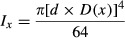
(9)

where *d* is the smallest diameter on the cone. The area moment of inertia with a circular shape varies with the *x*-axis. The area moment of inertia is proportional to the diameter and is smaller on the left-hand side, whilst larger on the right-hand side. Substituting Eqns 7–9 into Eqn 6 yields a nonlinear model of the cantilever beam with a cone shape; that is,


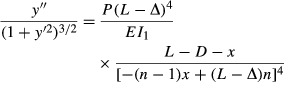
(10)

**Figure 2:**
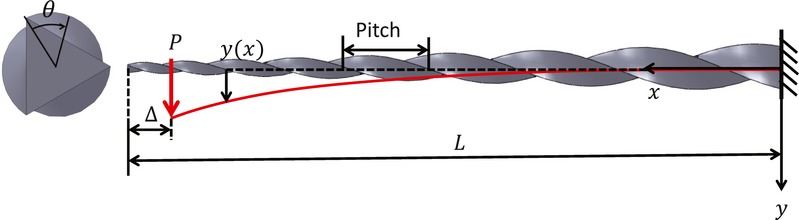
Screw pitch variation of rotary endodontic instrument subjected to a bending force *P*.

The complex shape of the instruments always includes screw and variable cross-section geometry. Figure 2 shows a rotary NiTi endodontic instrument with screw pitch variation, with a smaller pitch on the left-hand side and a larger pitch on the right-hand side. *L* denotes the total length of the beam and it will not change after deformation, *P* is a vertical concentrated load, and at the file tip *Δ* represents the horizontal displacement resulting from *P* action. The triangular area moments of inertia are written as


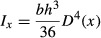
(11)


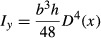
(12)


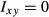
(13)

where *I*_*x*_ denotes the area moment of inertia about the *z*-axis, *I*_*y*_ the area moment of inertia about the *y*-axis, *I*_*xy*_ the product moment of inertia, *b* the triangle width and *h* the triangle height. *I*_*x*_ and *I*_*y*_ vary with *x*-axis and *I*_*xy*_ is zero that results from symmetric geometry. Moreover, the triangular shape of cross-section is inclined along with *x*-axis. Therefore, the area moment of inertia 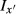 varies with respect to a set of inclined axis. The inclined area moment of inertia is formulated as (Boresi & Schmidt 2003)


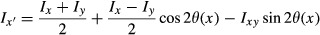
(14)

The value of *I*_*xy*_ will be zero with the symmetric geometry of cross-section. As depicted in Fig. 2, the angle of rotation is related to the pitch by


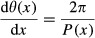
(15)

where pitch *P*(*x*) can be approximately expressed by


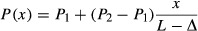
(16)

where *P*_1_ is the pitch value at the left end shown in Fig. 2, whilst *P*_2_ is the pitch value at the right end. The variation of pitch is linear and it varies with the *x*-axis. To obtain a nonlinear model of the instrument with triangular cross-section and screw pitch variation, substituting Eqn 8 and Eqn 11–16 into Eqn 6 yields


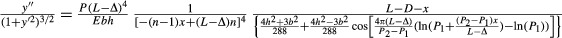
(17)

**Figure 3:**
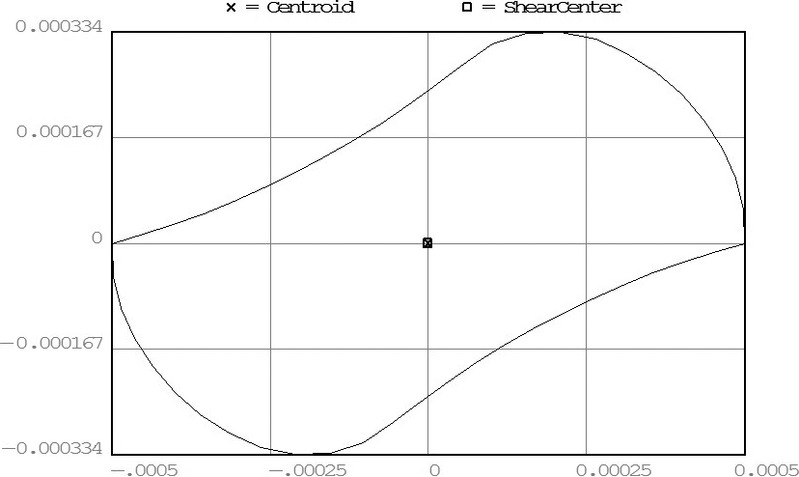
The cross-sectional geometry of NRT 0.06 taper instrument.

The nonlinear model of the instrument in terms of area moments of inertia is written as


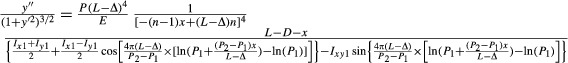
(18)

where *I*_*x*1_, *I*_*y*1_, and *I*_*xy*1_ denote area moments of inertia at the tip. This nonlinear model is suitable for any shape of cross-section. However, the values of area moments of inertia need to be calculated by formulas or software. Based on the foregoing derivation, this section presents analytical results for a RaCe size 25, 0.06 taper instrument (FKG; Dentaire Co., La Chaux-de-Fonds, Switzerland) and a Mani NRT size 30, 0.05 taper (MANI, Tochigi, Japan). RaCe has a simple geometry in cross-section (Yao *et al*. 2006), and NRT has been studied extensively (Kim *et al*. 2009, Pirani *et al*. 2011, Zhang *et al*. 2011). Moreover, RaCe has an axial symmetric geometry and NRT does not. The cross-sectional shape of the RaCe instrument is triangular, and its working length is 16 mm. The cross-sectional shape of the NRT instrument is a modified rectangle-based design and shown in Fig. 3. Its working length is 11 mm. The area moment of inertia at the tip is shown in Table 1.

**Table 1:** Area moment of inertias of RaCe 0.06 taper instrument and NRT 0.05 taper instrument

Type	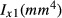	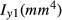	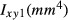
RaCe 0.06	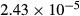	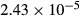	0
NRT	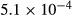	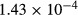	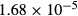

### Computer simulation for rotary NiTi endodontic instrument

Using finite element models, this study computed the stress distribution of NiTi instruments subjected to bending force caused by interaction with root canals. Dealing with the complex shape of rotary NiTi endodontic instruments is the main difficulty in simulation using finite element methodology. There are many rotary NiTi endodontic instruments in the market, and each manufacturer has different product sizes and shapes. This study deals with a RaCe size 25, 0.06 taper (FKG; Dentaire Co., La Chaux-de-Fonds, Switzerland) endodontic NiTi instrument that was subjected to bending loads, and its behaviours was investigated by a finite element software Ansys (ANSYS Inc., Canonsburg, PA, USA).

**Figure 4:**
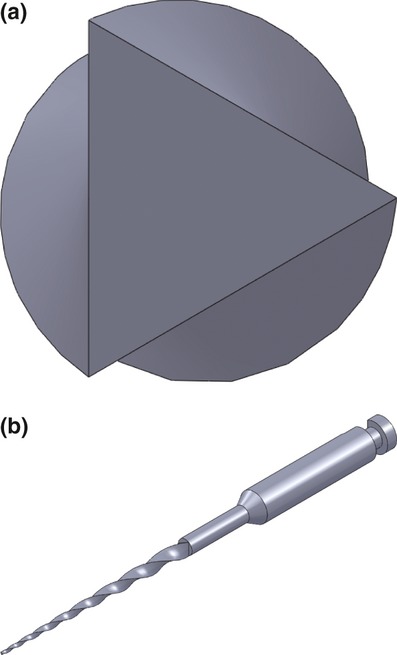
(a) Instrument top view that shows triangular cross-section; (b) isotropic view of the instrument.

Simulation of nonlinear analysis was used that allows large deformations without divergence. Young's modulus and Poisson's ratio of NiTi alloy were prescribed as 36 000 Mpa and 0.3, respectively (Kim *et al*. 2009; Table 2). A model of size 25, 0.06 taper RaCe rotary endodontic instrument of 16-mm working length was constructed. The model geometry depicted in Fig. 4 is a cone with triangle cross-section and screw pitch variation. Table 3 shows that the screw pitch and the cross-section diameter vary along the file axis.

**Table 2:** Material parameters of shape memory alloy

Parameter	Value
*E*	36 000 MPa
ν	0.3

**Table 3:** Pitch variation and diameter variation of 6% tapered RaCe instrument

	Parameter	

Position (mm)	Pitch (mm)	Diameter (mm)
0	0.88	1.2
16	2	0.24

**Figure 5:**
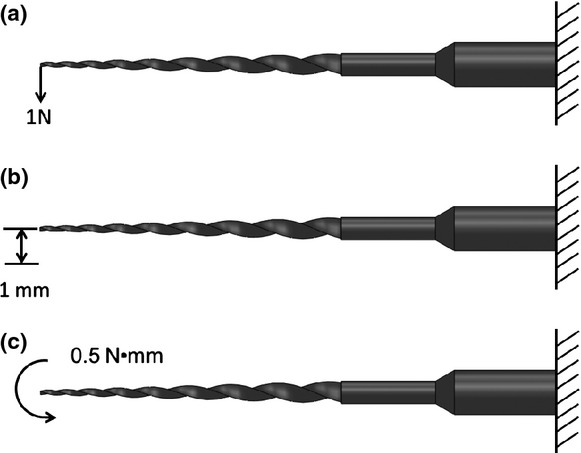
(a) Cantilever bending with a lateral force of 1 N applied to the tip of the instrument. (b) Cantilever bending until the tip was displaced by 1 mm. (c) Cantilever bending with bending moment of 0.5 N mm applied to the instrument.

As shown in Fig. 5, there are three kinds of loading conditions that are applied to the numerical model in finite element analysis:

1. Cantilever beam subjected to concentrated load, where bending is caused by a concentrated load of 1 N at the rotary instrument tip.
2. Cantilever beam subjected to vertical displacement, where bending is caused by a displacement of 1 mm at the instrument tip.
3. Cantilever beam subjected to bending moment, where bending is caused by a bending moment of 0.5 N mm on the instrument.

## Results

### Analytical result

**Figure 6:**
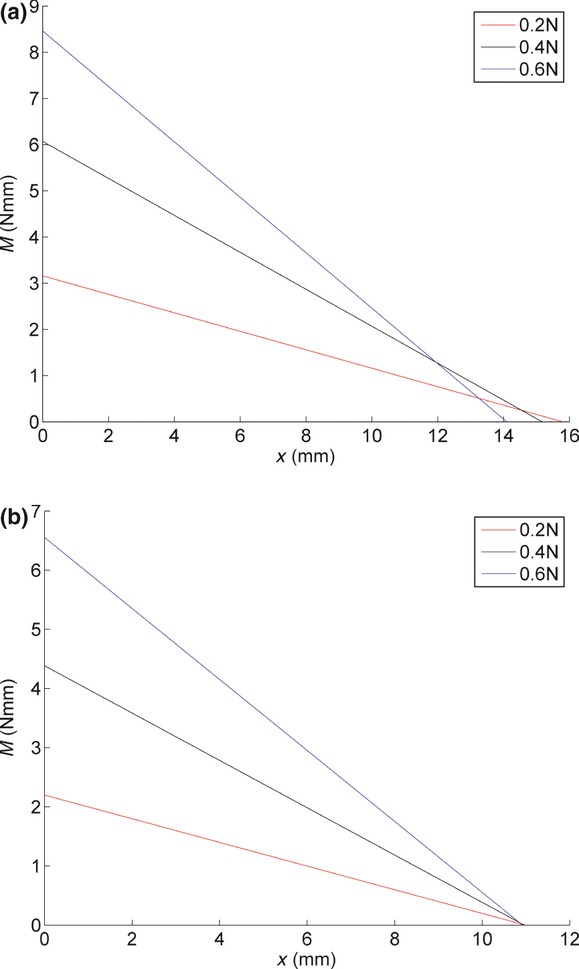
Comparison amongst bending moments of rotary endodontic instrument subjected to different lateral forces: (a) RaCe 0.06 taper and (b) NRT 0.05 taper.

**Figure 7:**
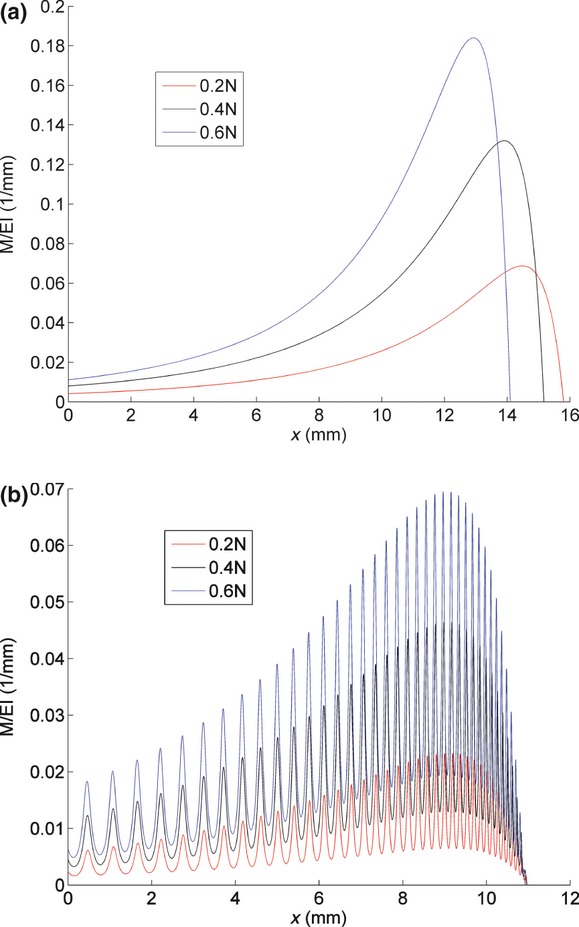
Comparison amongst curvatures of rotary endodontic instrument subjected to different lateral forces: (a) RaCe 0.06 taper and (b) NRT 0.05 taper.

Figure 6 depicts bending moment variation caused by a lateral force *P* shown in Fig. 2. The maximum bending moment *M* occurs at the origin *x* = 0 because the origin shown in Fig. 2 has the longest force arm. Comparing both instruments, the resulting bending moment of the RaCe instrument is larger than the NRT instrument under the same loading condition because the RaCe instrument has longer working length. The bending moment is proportional to both force arm and lateral force. Moreover, a larger applied force will lead to larger axial displacement *Δ* at the file tip depicted in Fig. 2, such that different force magnitudes lead to different arm lengths after deformation. Figure 7 depicts curvature variations of both instruments under different lateral forces.

**Figure 8:**
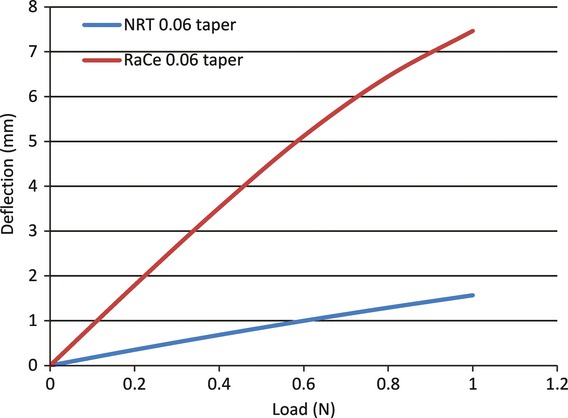
Comparison the results of deflection versus load between the RaCe 0.06 taper instrument and the NRT 0.05 taper instrument.

According to Eqn 3, curvatures are proportional to bending moments. Because bending moments are proportional to both lateral force and force arm, the curvature magnitude is proportional to the lateral force and will drop to zero at the free end, where the force arm becomes zero. The right-end positions of curvature curves in Fig. 7 vary with the horizontal displacement *Δ* of the file tip. Figure 7(b) shows that curvature curves of the NRT instrument wave along the *x*-axis, which is caused by the nonaxial symmetric geometry of the cross-section. According to Eqn 14, the pitch is inversely proportional to the fluctuation frequency, which equals to 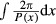. Finally, Fig. 8 compares deflection versus force results between both instruments.

### Numerical results

**Figure 9:**
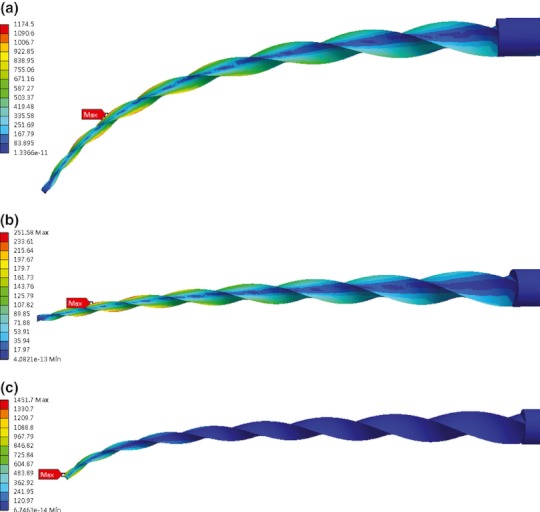
(a) von Mises stress distribution under 1 N concentrated force applied on the tip. (b) von Mises stress distribution under 1 mm displacement applied on the tip. (c) von Mises stress distribution under 0.5 N mm bending moment applied on the tip.

Figure 9 depicts results of a large deformation. When a concentrated load 1 N is applied at the tip, the end deflection of the instrument is 5.7 mm. Additionally, Fig. 9(a) reveals that a maximum von Mises stress of 1.17 Gpa is found 3.2 mm from the instrument tip. Furthermore, subjected to a 1 mm displacement at the tip, a maximum von Mises stress of 2.52 Gpa shown in Fig. 9(b) is found 1.9 mm from the instrument tip. Subjected to a bending moment of 0.5 N mm as shown in Fig. 9(c), a maximum von Mises stress of 1.45 Gpa is found at the instrument end.

**Figure 10:**
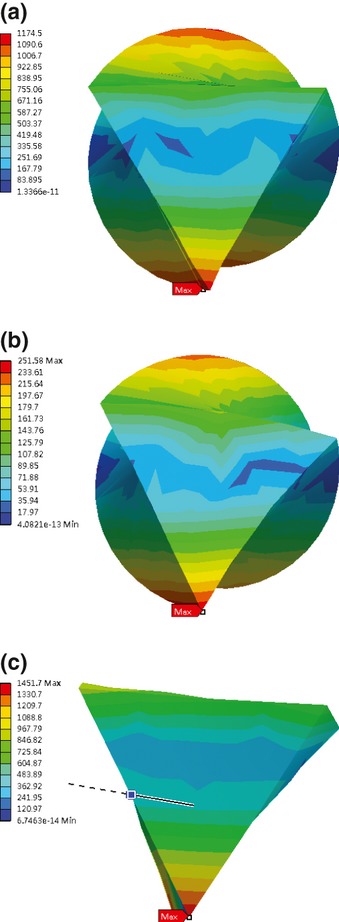
(a) von Mises stress distribution of cross-section view when lateral force of 1 N is applied to the tip. (b) von Mises stress distribution of cross-section view when the tip is displaced by 1 mm. (c) von Mises stress distribution of cross-section view when 0.5 N mm bending moment is applied.

Figure 10(a) shows the stress distribution of a cross-section view subjected to 1 N lateral force at the tip, and the maximum stress with 1.17 Gpa can be observed at the tip. Figure 10(b) shows the maximum stress distribution of a cross-section view with 2.52 Gpa when the tip is displaced laterally by 1 mm. The maximum stress also appears at the periphery of the instrument's cross-section. Figure 10(c) shows the maximum stress distribution of a cross-section view with 1.46 Gpa when a 0.5 N mm bending moment is applied. Under the three loading conditions, symmetry of stress distribution is not observed because the cross-section is not symmetric. The minimum stress always distributes on the neutral axis, whilst the maximum stress always occurs at triangle peaks that are most far away from the neutral axis.

**Figure 11:**
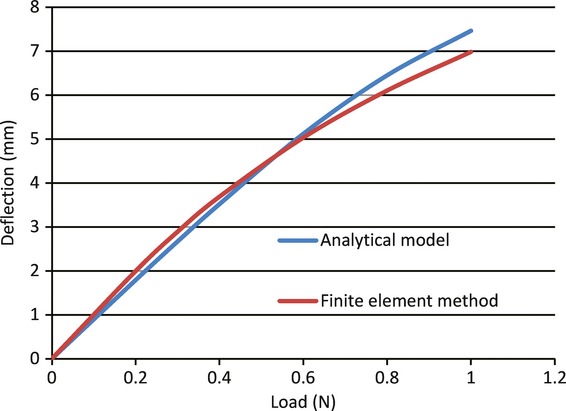
Comparison of deflection versus load results between analytical model and finite element method.

Figure 11 compares deflections resulting from analytical analysis and finite element analysis. Both results are consistent. In contrast to the finite element method, the present nonlinear model neglects shear force, which results in slight deviation in large loading condition.

The stress distribution of a cross-section view under 1 N lateral force applied to the tip is shown in Fig. 10(a), and the maximum stress with 1.17 Gpa can be observed at the tip. Figure 10(b) shows the maximum stress distribution of cross-section view with 2.52 Gpa when the tip was displaced by 1 mm. The maximum stresses also appear at the periphery of the instrument's cross-section. Figure 10(c) shows the maximum stress distribution of a cross-section view with 1.46 Gpa when a 0.5 N mm bending moment is applied. Under the three kinds of loading conditions, symmetry of stress distribution is not observed because the cross-section of the instrument is not symmetric. The minimum stress always distributes on the neutral axis, whilst the maximum stress always occurs at a peak in the triangle that is most far away from the neutral axis.

## Discussion

Compared with the NRT instrument, the curvature of the RaCe instrument is larger under the same loading condition. According to Eqn 1 and 2, the position of maximum curvature is also the place where the maximum stress and strain occur. Accordingly, the flexibility of RaCe instruments is greater than NRT instruments. Greater flexibility allows the instrument to pass along the curve of root canals more effectively, whereas undesirable deformation is easier to generate at high rotational speeds.

Comparing the three loading conditions in a computer simulation, revealed that the bending moment caused stress to be more concentrated on the tip because the moment magnitude is proportional to the distance between tip and position of cross-section when the force or displacement is applied at the tip. However, it was constant along the position of cross-section when the bending moment was applied. The bending moment has the minimum low stress area. Figure 11 compares deflections resulting from analytical analysis and finite element analysis. Eqn (31) in Appendix S2 shows the nonlinear model of the instrument, and the analytical results are presented by Eqn (21) in Appendix S1. The result of the analytical model and finite element method are close to each other. Compared with the finite element method, the present nonlinear model neglects shear force, which results in slight deviation in large loading condition.

## Conclusion

The proposed analytical model has been compared with and validated by numerical results in analysing bending deformation of NiTi instruments. The analytical model is applicable to any cross-sectional geometry. Therefore, it is useful to design and analyse rotary NiTi endodontic instruments. Table 4 summarizes the advantages and disadvantages between the finite element method and the present method. For mechanic analysis of rotary NiTi endodontic instruments, finite element analysis is popular because it can handle complex geometry and yields results in detail, but the procedure may be complex and time consuming. It may also be expensive to build finite element models. By contrast, although analytical derivation looks complicated and is often difficult to understand, analytical results can be used in real-time implementation for online detection, monitor and feedback control of rotary NiTi endodontic instruments.

**Table 4:** Advantages and disadvantages between the finite element method and present method

Method	Advantage	Disadvantage
Finite element method	Detailed and accurate results	The models may be difficult and expensive to build
	Handling complex geometries	Complex and time-consuming procedure
Present method	Convenient and effective procedure	Formulations can be complicated
	Low cost	Not easy to deal with complex geometry problem

## Additional Supporting Information may be found in the online version of this article:

**Appendix S1.** Method of solving the nonlinear differential equation.

**Appendix S2.** Integration of the Euler–Bernoulli nonlinear differential equation.
