# Supplementary material for: Study on bending behaviour of nickel–titanium rotary endodontic instruments by analytical and numerical analyses
Source: Int Endod J. 2012 Nov 22;46(4):379–88. doi: 10.1111/iej.12025 (PMC3617459; doi:10.1111/iej.12025)
Supplement: Supplementary file 1 [file iej0046-0379-SD1.docx]

**Appendix A: Method of Solving the Nonlinear Differential Equation**

(6)

where

Integrating Eqn (6) and substituting boundary condition with at yields

(19)

where is obtained from integrating . And the procedures of integration is described in Appendix B. Rearranging Eqn (19) gives

(20)

Integrating Eqn (20) yields the instrument deflection

(21)

The horizontal displacement Δ can be solved from

(22)

**Appendix B: Integration of the Euler-Bernoulli Nonlinear Differential Equation**

In order to integrate Eqn (6), variables are first altered. Let

$y^{''}=p^{'}$ (23)

(24)

Accordingly, Eqn (6) becomes

(25)

where

(26)

By multiplying both sides of Eqn (25) by dx and integrating once, we can find

(27)

The arc slope is written as

(28)

where

(29)

Thus,

(30)

It follows from Eqn (28) and Eqn (29) that

(31)

(32)

Substituting Eqn (29) and Eqn (30) into Eqn (27) yields

(33)

Rearranged Eqn (33) becomes

(34)

Finally, the solution of Eqn (34) is written as

(35)

Substituting Eqn (32) into Eqn (35) yields

(36)

where is an integration constant, which can be determined by a boundary condition. Substituting Eqn (24) into Eqn (36) leads to

(37)

Eqn (37) can be rewritten as an equation of slope

(38)

Finally, integrating Eqn (38) yields the equation of deflection.

(39)
